# *AXL*-mRNA Overexpression in Size-Based Enriched Circulating Tumor Cells as a Potential Biomarker for Anti-AXL Targeted Therapies in Non-Small-Cell Lung Cancer

**DOI:** 10.3390/cancers18111759

**Published:** 2026-05-28

**Authors:** Aliki Ntzifa, Elena Themistokli, Areti Strati, Martha Zavridou, Emilia Tsaroucha, Aggeliki Sfika, Amanda Psyrri, Ioanna Balgouranidou, Athanasios Kotsakis, Vassilis Georgoulias, Evi Lianidou

**Affiliations:** 1 Analysis of Circulating Tumor Cells Laboratory, Laboratory of Analytical Chemistry, Department of Chemistry, National and Kapodistrian University of Athens, 15771 Athens, Greece; elenathemistokli@gmail.com (E.T.); astrati@uoa.gr (A.S.); mzavridou@chem.uoa.gr (M.Z.); 2 7th Department of Pulmonary Diseases, “Sotiria” General Hospital of Athens, 11527 Athens, Greece; emilygeola@yahoo.gr; 3 Oncology Unit, 2nd Department of Surgery, Aretaieio Hospital, Medical School, National and Kapodistrian University of Athens, 11528 Athens, Greece; angelikisfika@hotmail.com; 4 Medical Oncology Unit, 2nd Department of Internal Medicine, “Attikon” General Hospital of Athens, 12462 Athens, Greece; dpsyrri@med.uoa.gr; 5 Department of Medical Oncology, University General Hospital of Alexandroupolis, Medical School, Democritus University of Thrace, 68100 Alexandroupolis, Greece; ibalgkou@med.duth.gr; 6 Department of Medical Oncology, University General Hospital of Larissa, 41334 Larissa, Greece; thankotsakis@uth.gr; 7 Hellenic Oncology Research Group (HORG), 11471 Athens, Greece; georgulv@otenet.gr

**Keywords:** *AXL*, liquid biopsy, CTCs, NSCLC, osimertinib, immunotherapy

## Abstract

**Background**: AXL, a tyrosine kinase receptor, is involved in epithelial-to-mesenchymal transition (EMT), cell survival, invasion, metastasis, and resistance to EGFR-TKIs and immune checkpoint inhibitors (ICIs). Recent clinical studies have shown promising results in NSCLC patients treated with AXL inhibitors. **Methods**: We evaluated *AXL*-mRNA overexpression in CTC fractions of NSCLC patients under osimertinib treatment (Group A, *n* = 39), (collected in the context of a multicenter Phase II clinical study (ClinicalTrials.gov number: NCT02771314) or immunotherapy (Group B, *n* = 116) at different time points collected in the context of a prospective, multicenter study (ClinicalTrials.gov number: NCT04490564). Size-based CTC enrichment (Parsortix, CelLBx Health, Guildford, UK) was used, and *AXL*-mRNA overexpression was evaluated using RT-qPCR. **Results**: In Group A, *AXL*-mRNA overexpression in CTC fractions was detected in 5/39 (12.8%) patients’ samples at baseline, in 5/31 (16.1%) after one cycle of treatment, in 7/79 (8.9%) during treatment and in 4/32 (12.5%) at progression of disease (PD). In Group B, *AXL*-mRNA overexpression in CTC fractions was detected in 8/116 (6.9%) samples before immunotherapy, in 8/71 (11.3%) after three or four cycles, while no *AXL* transcripts were detected at PD. **Conclusions**: Our results indicate that *AXL*-mRNA overexpression in CTC fractions deserve to be further evaluated through larger clinical studies as a potential biomarker for anti-AXL targeted therapies in NSCLC.

## Simple Summary

AXL, a tyrosine kinase receptor, is involved in epithelial-to-mesenchymal transition (EMT), cell survival, invasion, metastasis, and resistance to tyrosine kinase inhibitors of epidermal growth factor receptor (EGFR-TKIs) and immune checkpoint inhibitors (ICIs). Recent clinical studies have shown promising results in non-small-cell lung cancer (NSCLC) patients treated with AXL inhibitors. In the present study, we evaluated, for the first time, *AXL*-mRNA overexpression in size-based enriched circulating tumor cell (CTC) fractions in longitudinal liquid biopsy samples from two independent groups of NSCLC patients: (a) under osimertinib treatment and (b) during immunotherapy. Our results indicate that *AXL*-mRNA overexpression in CTC fractions deserves to be further evaluated through larger clinical studies as a potential biomarker for anti-AXL targeted therapies in NSCLC.

## 1. Introduction

During the last few decades, the treatment landscape of non-small-cell lung cancer (NSCLC) has been successfully altered. The development of immune checkpoint inhibitors (ICIs) has revolutionized the NSCLC treatment approach leading to improved clinical outcomes of cancer patients [1,2]. Specific targeted therapies, including those against somatic mutations found in the tyrosine kinase (TK) domain of epidermal growth factor receptor (EGFR), have also shown improved efficacy to *EGFR*-mutant NSCLC patients compared to conventional chemotherapeutic agents [3]. However, there is a common challenge that patients need to overcome; treatment resistance remains a major obstacle, limiting long-term response to treatments and prolonged survival rates [4]. Tumor heterogeneity, driver mutations, tumor microenvironment, and activation of alternative signaling pathways are some of the significant factors of resistance. The detection of novel biomarkers and the development of new anti-target therapies are common strategies to overcome resistance [4,5].

AXL, a member of the TAM (TYRO3, AXL, and MERTK) receptor tyrosine kinases (RTKs) family, has gained attention through its versatile roles in cell proliferation and survival, metastasis, tumor progression and treatment resistance [6]. Its implication in epithelial-to-mesenchymal transition (EMT), renders it a significant EMT marker in various types of cancer [7]. *AXL* is found to be highly expressed in NSCLC and more specifically its overexpression has been linked to the *EGFR* signaling pathway [8]. One of the alternative signaling mechanisms of acquired resistance to osimertinib is the bypass activation of AXL [9]. Moreover, there is a growing body of evidence that *AXL* plays a key role in resistance to immunotherapy in NSCLC [10].

Liquid biopsy is now an established minimal invasive approach that has revolutionized the management of NSCLC patients, through integration of circulating tumor DNA (ctDNA) analysis into clinical practice to facilitate personalized treatment [11,12]. In liquid biopsy, CTC analysis has a unique advantage over ctDNA analysis, since it can provide highly important information at the protein and gene expression level, for important therapeutic targets such as *PD-L1*, *AR-V7*, and *AXL*. Through molecular characterization, CTC analysis unveils important information on the molecular heterogeneity of disease and tumor clonal evolution, leading to the identification of resistance mechanisms and the development of novel therapeutic approaches [13]. Previous recent studies have shown that *AXL* is overexpressed in CTCs in advanced NSCLC [14] and also in metastatic breast cancer, suggesting liquid biopsy as a promising approach during AXL inhibition [15]. *AXL* tissue expression in combination with CTC enumeration could predict patient outcomes in resected lung adenocarcinomas [16]. Our group was the first to report a possible EMT role of *AXL* in the development of acquired EGFR-TKI resistance based on *AXL*-mRNA expression levels in CTC fractions of NSCLC patients [17,18].

In a recent review, it is reported that AXL inhibition could be a promising therapeutic intervention in *EGFR*-mutant NSCLC or during immunotherapy based on preclinical and clinical studies [19]. At present, several clinical trials are examining the effectiveness of AXL-inhibitors combined with EGFR-TKIs or ICIs [19]. Bemcentinib is the only selective small-molecule AXL inhibitor that obtained US FDA fast-track designation for treating advanced or metastatic NSCLC patients when used alongside a PD-L1 inhibitor in the phase 2 BGBC008 trial (NCT03184571) [20].

Based on these recent findings, in the present study, we evaluated, for the first time, whether *AXL* could be a novel CTC biomarker and therapeutic target in NSCLC. To this end, we evaluated, for the first time, *AXL*-mRNA overexpression in size-based enriched CTC fractions in longitudinal liquid biopsy samples from two independent groups of NSCLC patients, (a) under osimertinib treatment and (b) during immunotherapy. We further investigated the role of *AXL*-mRNA overexpression in CTC fractions as a potential biomarker for anti-AXL targeted therapies in NSCLC.

## 2. Materials and Methods

### 2.1. Patients

In the present study, clinical samples from two independent cohorts of NSCLC patients were analyzed: (a) under osimertinib treatment (Group A) and (b) under immunotherapy (Group B). Patients and healthy donors (HD) gave their written informed consent to participate in two different clinical studies, respectively. Both studies were conducted in accordance with the Declaration of Helsinki.

Group A: Thirty-nine patients with histologically or cytologically confirmed *EGFR*-mutant lung adenocarcinomas received second-line osimertinib (AZD9291; Astra Zeneca, Cambridge, UK) as part of a multicenter Phase II clinical trial (ClinicalTrials.gov number: NCT02771314, registration date: 13 May 2016) and EudraCT number: 2016-001335-12, registration date: 13 April 2016) organized by the Hellenic Oncology Research Group (HORG). The research has received approval from the National Drug Administration of Greece (EOF), the National Ethics Committee (35/00-03/16, 35/03-11/16), and the Institutional Ethical Committees of the centers participating in HORG. At the start of this clinical study, osimertinib was given as a second-line therapy to NSCLC patients with *EGFR* mutations, following the existing guidelines at that time.

Group B: Peripheral blood (PB) samples from 116 patients with NSCLC were collected at five clinical centers in Greece as part of a prospective, multicenter research study (ClinicalTrials.gov identifier: NCT04490564, registration date: 28 July 2020). Eligibility criteria for patients with metastatic non-small-cell lung cancer (NSCLC): starting PD-1 inhibitors typically required male and female individuals aged 18 and above. The Ethics committees of all involved institutions granted approval for the study: Attikon University Hospital (7th meeting of the Scientific Advisory Board 10 September 2019), “SOTIRIA” General Hospital of Athens (19261, 28 August 2019); Aretaieion University Hospital, (137/28/11/19); General University Hospital of Alexandroupolis, (6th regular meeting of the Scientific Advisory Board, 19 June 2019).

### 2.2. Collection of PB Samples and Processing

PB was obtained in EDTA tubes after rejecting the initial 5 mL of blood draw to prevent contamination from skin epithelial cells. In Group A, 15 mL of PB were collected from NSCLC patients with *EGFR* mutations at different intervals as follows: (a) 39 samples at baseline, prior to starting treatment with osimertinib; (b) 31 samples after the first cycle of therapy; (c) 79 samples approximately every 3 months during treatment; and (d) 31 samples upon disease progression (PD). In Group B, PB (10 mL) was obtained from NSCLC patients at 3 distinct time points as follows: (a) 116 samples prior to immunotherapy, (b) 71 samples following 3 or 4 cycles of ICIs, and (c) 10 samples at PD. PB samples collected from a control group consisting of 46 HDs were processed and analyzed using the same experimental procedure. All preanalytical conditions for sample collection, transportation and storage conditions were strictly controlled and standardized, as the ACTC lab is ISO-15189 certified [21].

### 2.3. CTC Enrichment

PB samples were centrifuged at 530× *g* (10 min at room temperature); the supernatant plasma was extracted and stored at −70 °C for subsequent analysis. In the remaining pellet of blood cells, an equal volume of phosphate-buffered saline (PBS; pH 7.3) was added to the plasma that was removed. Then, the FDA-cleared Parsortix™ (CelLBxHealth, Guildford, UK) device was used for CTC enrichment, using a 6.5 μm separation cassette. The harvested cells were collected in a final volume of 210 μL PBS.

### 2.4. Total RNA Extraction—cDNA Synthesis

Following CTC enrichment, TRIzol LS reagent (Thermo Fisher Scientific, Waltham, MA, USA) was used for total RNA extraction from the size-based enriched CTC fractions in RNAase free conditions. RNA was dissolved in Ambion RNA Storage Solution (Thermo Fisher Scientific), quantified using a NanoDrop ND-1000 UV–Vis Spectrophotometer (Thermo Fisher Scientific), and maintained at −70 °C. A High-Capacity RNA-to-cDNA kit (Thermo Fisher Scientific) was used for cDNA synthesis in a total volume of 20 μL, following the manufacturer’s instructions and previously outlined methods [17].

### 2.5. In Silico Design of Primers and Probes

In silico design for the *AXL* primers and probe was performed using Primer Premier 5.0 software (Premier Biosoft, Palo Alto, CA, USA). The Basic Local Alignment Search Tool (BLAST), version 2.11.0 (NCBI, NIH) was used for the design of primers and probes to prevent primer–dimer formation, false priming locations, hairpin structure formation, and hybridization with genomic DNA. The sequences of primers and probes are the following: Forward primer: 5′-AGAAGGAGACCCGTTATGGAGA-3′; Reverse primer: 5′-ATGCCCAGGCTGTTCAAGG-3′; Taqman probe: 5′-FAM-CTTCAGTGGTCCGACGACTGTAGGACTT-BHQ-3′.

### 2.6. RT-qPCR

RT-qPCR was performed to evaluate *AXL* mRNA expression, as follows: the amplification reaction mixture for *AXL* contained 4 μL of the PCR synthesis buffer (5×, Promega, Madison, WI, USA), 1.2 μL MgCl2 (25 Mm, Promega), 0.2 μL dNTPs (10 mM, Thermo Fisher Scientific), 0.1 μL Hot-Start DNA polymerase (5 U/μL, Promega), 0.3 μL of forward and reverse primer (10 μΜ, IDT, Coralville, IA, USA), 0.83 μL hydrolysis probe (3 μM, IDT) and DEPC–H_2_O, to a final volume of 10 μL. PCR cycling conditions for *AXL* were: 95 °C for 2 min, 45 cycles of 95 °C for 10 s, annealing at 58 °C for 20 s, extension at 72 °C for 20 s, and a final cooling cycle at 40 °C for 30 s.

The expression of *Beta-2 microglobulin* (*B2M*) was used as a reference gene for ensuring the presence of amplifiable material in all samples and for normalization of *AXL* mRNA expression, as previously described [17]. All RT-qPCR assays were performed in the cobas z480 system (Roche Molecular Systems, Inc., Pleasanton, CA, USA).

### 2.7. Normalization of RT-qPCR Data for AXL Expression

RT-qPCR data for *AXL* expression were normalized with respect to *B2M* expression in the same cDNAs, using the 2^−ΔΔCq^ method [22]. CTCs were enriched using the size-based Parsortix technology (CelLBx, Guildford, UK); to correct the background noise due to the co-isolation of peripheral blood mononuclear cells (PBMC), present in all CTC enrichment systems, PB from a group of HDs was analyzed in exactly the same way as the patients’ and the results were used to establish a cut-off value through normalization of *AXL* expression with respect to *B2M* expression. The inclusion of a healthy donor group is very important, since CTCs are enriched through Parsortix and not isolated as pure cancer cells; therefore, every sample contains also a number of PBMCs, and this background should be taken into account. The cut-off value for *AXL* transcripts was calculated as the mean value of signals derived in the HDs group plus two standard deviations (2SD), as previously described [23]. Based on this approach, a clinical sample is defined as *AXL* overexpressed (*AXL*-positive) based on the fold change in *AXL* expression in the CTC fraction with respect to the corresponding PBMC fraction in the group of HDs.

### 2.8. Statistical Analysis

Statistical analysis was conducted utilizing IBM SPSS Statistics for Windows, version 31.0 (IBM Corp., Armonk, NY, USA). The McNemar test was used to evaluate the concordance of gene expression across various time points. Every *p*-value is two-tailed. A *p*-value of less than 0.05 is considered statistically significant.

## 3. Results

**Figure 1:**
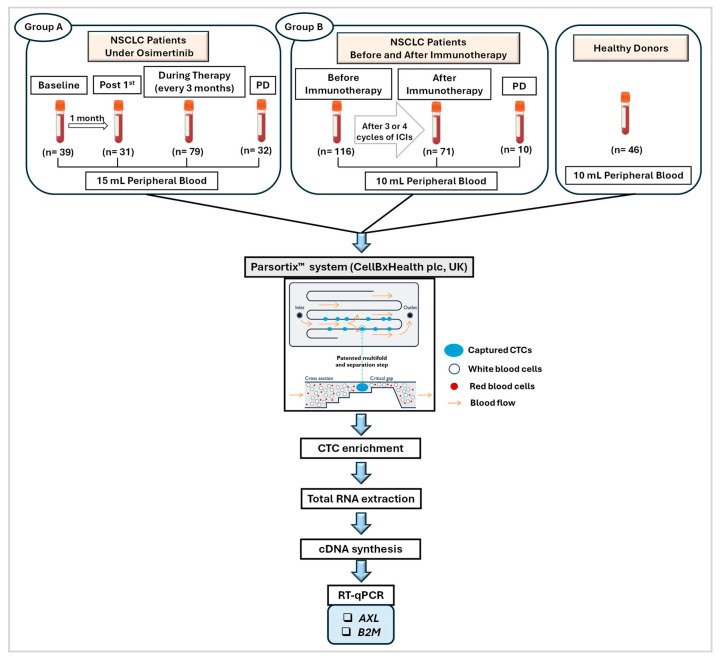
Experimental flowchart of the study.

The outline of the study is shown in Figure 1.

### 3.1. AXL-mRNA Expression Levels in Size-Based Enriched CTC Fractions

*AXL* transcripts were detected at very low levels in PB samples from HDs (*n* = 46) that were analyzed in the same way. Thus, the 2^−ΔΔCq^ method was used for normalizing *AXL*-mRNA expression levels in patients’ samples with respect to the expression of *B2M* as reference gene, as previously described [17,22,23,24]. Since the total PB volume used for CTC enrichment was different in these two groups, the samples for the control group of HDs were analyzed in exactly the same way, using 15 mL PB for Group A and 10 mL PB for Group B. For Group A, the cut-off value for *AXL* transcripts was calculated as the mean value of signals derived in the HDs group (*n* = 10), plus two standard deviations (2SD), as previously described (15 mL PB, cut-off: 2^−ΔΔCq^ = 1.55) [17]. The same approach was used for Group B analyzing PB samples from 36 HDs (10 mL PB, cut-off: 2^−ΔΔCq^ = 1.80).

### 3.2. AXL-mRNA Overexpression in Size-Based Enriched CTC Fractions of NSCLC Patients Under Osimertinib

**Figure 2:**
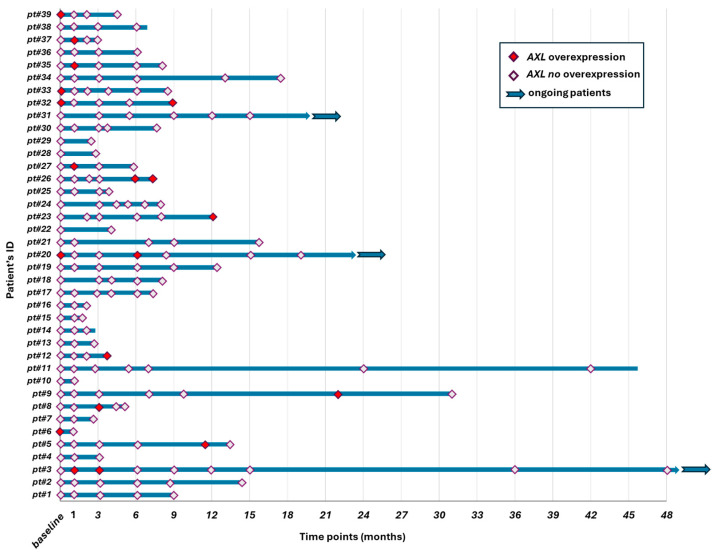
Group A: *AXL*-mRNA overexpression in size-based enriched CTC fractions with respect to *B2M* expression of NSCLC patients at different time points during osimertinib treatment.

Group A: NSCLC patients were longitudinally monitored one month after osimertinib initiation and then approximately every 3 months and until PD. The expression of *AXL* was also evaluated at these different time-points (Figure 2). In most patients, *AXL*-mRNA overexpression was detected only at a single time point; whereas, in some cases (Pt#3, Pt#20, Pt#26, and Pt#32) was detected at two distinct time points during treatment. *AXL*-mRNA overexpression in CTC fractions was detected in 5/39 (12.8%) patients’ samples at baseline; whereas, after one cycle of treatment, *AXL*-mRNA overexpression was detected in 5/31 (16.1%) samples. *AXL*-mRNA overexpression in CTC fractions was detected in 7/79 (8.9%) samples during various stages of treatment and in 4/32 (12.5%) samples at PD. At the time of analysis, three patients were still under osimertinib treatment (ongoing).

### 3.3. Longitudinal Monitoring of AXL-mRNA Overexpression in CTC Fractions During Osimertinib

**Figure 3:**
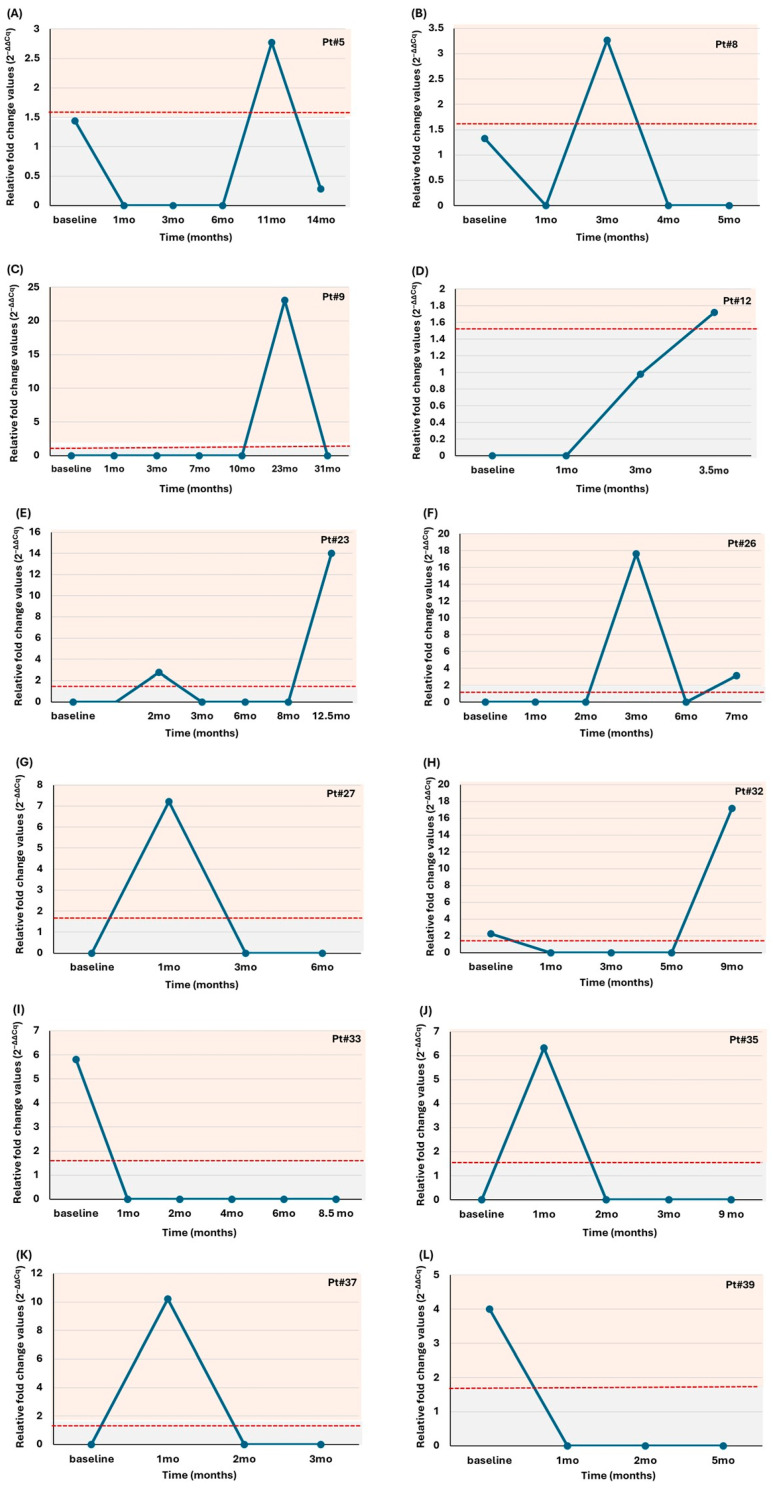

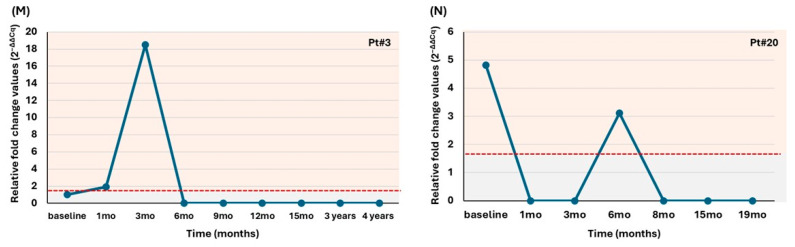
Relative fold change values of *AXL*-mRNA overexpression in CTC fractions of NSCLC patients who presented PD (**A**–**L**) over time and of two ongoing NSCLC patients (**M**,**N**) over time (red line depicts cut-off value: 2^−ΔΔCq^ = 1.55, reddish area: above cut-off value, grayish area: below cut-off value).

Next, we assessed the levels of *AXL*-mRNA overexpression and their deviations over time during osimertinib treatment in a subset of 12 NSCLC patients that reached PD (Figure 3A–L) and in two NSCLC patients that were still under osimertinib treatment (ongoing) at the time of analysis (Figure 3M,N).

*AXL*-mRNA overexpression was detected in CTC fractions of patients Pt#32, Pt#33, Pt#39 at baseline; whereas, in patients Pt#27, Pt#35, and Pt#37 *AXL*-positive CTC fractions were detected after one cycle of treatment (within the first month after osimertinib initiation). CTC fractions of Pt#12, Pt#23, Pt#26 and Pt#32 were positive for *AXL*-mRNA overexpression at PD. In Pt#9, *AXL*-positive CTC fractions were detected after 23 months of osimertinib treatment and the patient progressed almost 8 months later. *AXL*-mRNA overexpression was detected in CTC fractions of Pt#8 and Pt#26 after 3 months of osimertinib treatment and both these patients progressed after almost 4 months (Figure 3A–L). Longitudinal monitoring of ongoing NSCLC patients did not reveal any specific pattern of *AXL*-mRNA overexpression. More specifically, Pt#3 had *AXL*-positive CTC fraction after 3 months of treatment and subsequently the levels of *AXL*-mRNA overexpression were below the cut-off value. *AXL*-mRNA overexpression was detected in Pt#20 before treatment (baseline) and then after six months of therapy at lower levels. Between these two time-points, after six months and at the time of analysis, no *AXL*-positive CTC fraction was detected (Figure 3M,N).

### 3.4. AXL-mRNA Overexpression in Size-Based Enriched CTC Fractions of NSCLC Patients Before and After Immunotherapy

Group B: *AXL*-mRNA overexpression in CTC fractions was detected in 8/116 (6.9%) patients prior to treatment, in 8/71 (11.3%) after three or four cycles of treatment with ICIs, while no *AXL* transcripts were detected in any of the 10 patients at the time of PD. *AXL*-mRNA overexpression was not detected in matched samples before and after immunotherapy for any of the NSCLC patients. No statistically significant differences were observed between the time points (before and after immunotherapy: McNemar test, *p* = 0.227).

**Figure 4:**
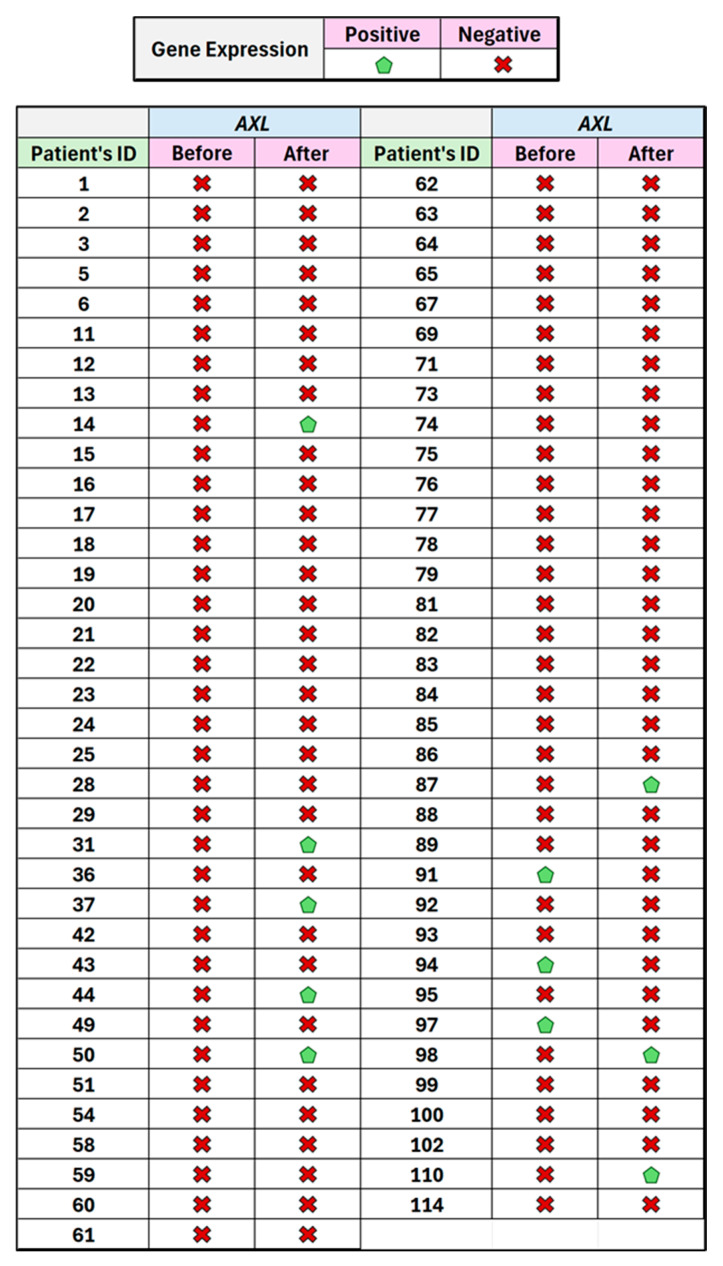
Group B: Direct comparison of *AXL*-mRNA overexpression detected in CTC fractions before and after immunotherapy for 71 metastatic NSCLC patients.

**Figure 5:**
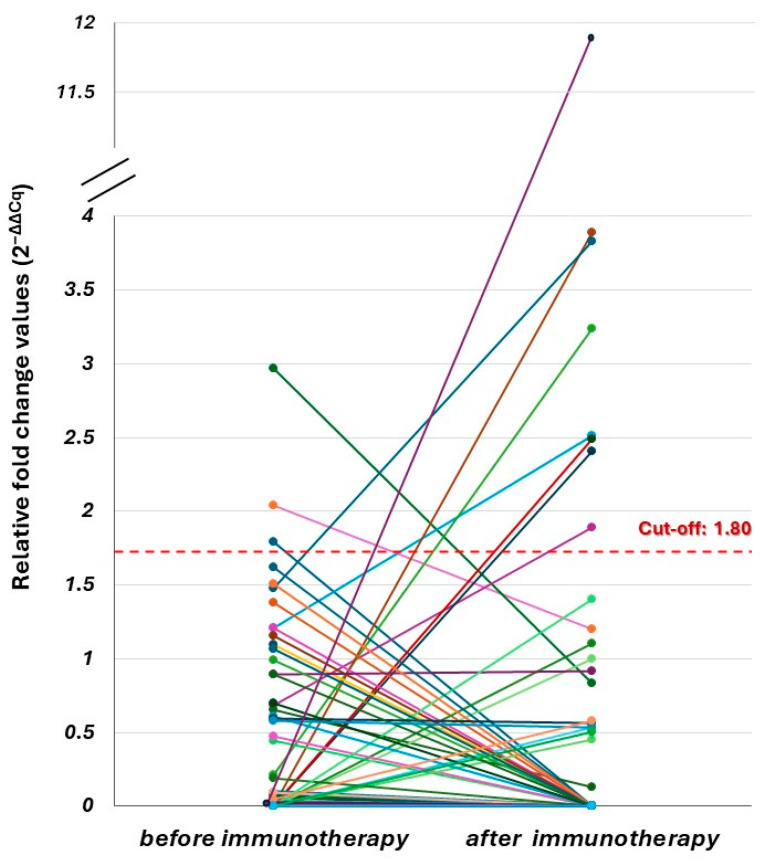
Relative fold change values of *AXL*-mRNA overexpression in CTC fractions from metastatic NSCLC patients in paired samples (*n* = 71) before and after immunotherapy (each line represents an individual patient, red dashed line depicts cut-off value: 2^−ΔΔCq^ = 1.80).

Direct comparison of *AXL*-mRNA overexpression detected before and after immunotherapy for 71 NSCLC patients revealed that three patients (Pt#91, Pt#94, Pt#97) had relative fold change values for *AXL*-mRNA overexpression detected in CTC fractions below the cut-off value after immunotherapy compared to the higher levels detected before immunotherapy. Conversely, *AXL*-mRNA overexpression in CTC fractions was detected in higher levels after immunotherapy for eight patients (Pt#14, Pt#31, Pt#37, Pt#44, Pt#50, Pt#87, Pt#98, and Pt#110). Pt#110 had the highest difference in levels of *AXL*-mRNA overexpression between the two time-points (Figure 4 and Figure 5).

## 4. Discussion

In this study, we evaluated *AXL*-mRNA overexpression in size-based enriched CTC fractions from NSCLC patients under osimertinib treatment and for the first time in NSCLC patients before and after immunotherapy.

Since the first report indicating *AXL* kinase as a novel mechanism of acquired resistance in *EGFR*-mutant NSCLC [25], many other preclinical and clinical studies have confirmed these primary data and further highlighted the role of *AXL* in EMT and drug resistance in NSCLC [26,27]. Numerous studies have reported that *AXL* confers resistance to EGFR-TKIs either as a bypass signaling pathway or by disrupting feedback signals [19,28]. Recently, the interest shifted to treating *EGFR*-mutant NSCLC patients with third-generation EGFR-TKIs, such as osimertinib [29]. In vitro and in vivo experiments have identified *AXL* as an important resistance mechanism to osimertinib. These results were also confirmed by data showing the effective outcomes of AXL inhibition in drug resistant cells [8,9,30,31]. *AXL* transcript levels significantly correlated with the tumor mutations burden and the emergence of the T790M, as this was also shown by other groups [32]. Unlike other driver genes, *AXL* expression is mediated through alternative mechanisms rather than gene mutations or amplifications [33]. Many different signaling pathways have been proposed to be involved in AXL-mediated resistance to EGFR-TKIs [30,32,34,35,36,37,38,39].

Our study was the first to detect *AXL*-mRNA overexpression in CTC fractions of NSCLC patients under osimertinib treatment [17]. In the present study, in Group A, an increased number of patients was detected with *AXL*-positive enriched CTCs after first cycle of treatment (16.1%) compared to baseline or later cycles of treatment and PD. Our results confirm our previous observation, although a smaller number of NSCLC patients was analyzed then [17]. Taniguchi et al. have shown that *AXL* overexpression was detected either in the initial or in the tolerant phase of cells treated with osimertinib [9]. Longitudinal monitoring of ongoing NSCLC patients during osimertinib, after one month of treatment and before PD, did not reveal any specific pattern of *AXL*-mRNA overexpression in CTC fractions. Compared to baseline, post 1st cycle and PD data, *AXL*-mRNA overexpression was detected in relatively low percentages (8.9%) during the follow-up of patients which can be explained by the effect of osimertinib on CTCs during treatment, as was shown in our previous study [18]. In addition, following the NSCLC patients during osimertinib treatment through *AXL*-mRNA overexpression in CTC fractions, there was a heterogeneous pattern of gene expression among them, further highlighting the significance of personalized treatment.

CTCs act as precursors of metastasis and their analysis unravels oncogenic alterations related to metastasis or to treatment resistance [13,40]. Moreover, CTC analysis offers a great potential for unveiling the heterogeneous tumor profile and novel resistance mechanisms in NSCLC [41,42]. In *EGFR*-mutant NSCLC, distinct CTC subpopulations exhibit a more aggressive or stem-like character [17,43,44]. Thus, the development and use of “size-based” CTC enrichment technologies like Parsortix, was proved to be optimal for detecting mesenchymal CTCs in NSCLC [14,17,24]. It has been demonstrated that AXL overexpression is also involved in EMT-mediated resistance to EGFR-TKIs [7,27]. Accordingly, it has been found that AXL inhibition can reverse EMT by resensitizing cells [26,27]. The most frequently observed CTC marker related to EMT is vimentin (*VIM*) demonstrating the key role of EMT in EGFR-TKI resistance [17,43,44,45]. Upregulation of *AXL* is often observed concurrently with the expression of *VIM* whereas *VIM* knockdown decreased *AXL* expression and restored erlotinib sensitivity [25]. Conversely, *AXL* inhibition did not affect EMT or *VIM* levels, suggesting that *AXL* upregulation in EGFR-resistant NSCLC cells may occur downstream of EMT [46]. EMT also modulates cancer cells by conferring metastatic capacity and transforming them into more aggressive cells with the aim of migrating to a potential metastatic site [47]. *AXL* overexpression was detected concurrently with *VIM*-positive CTCs, further enhancing the role of *AXL* in EMT and its implication in acquired EGFR-TKI resistance [14,17,25]. In our previous study, we have examined in parallel *AXL* and *VIM* overexpression in a small number of size-based enriched CTC fractions. According to our findings, in 11/81 (13.6%) samples, *VIM* was co-expressed with *AXL*—1/81 (1.2%) was only *AXL*-positive, and 41/81 (50.6%) were only *VIM*-positive [17]. This could possibly be explained by the fact that CTCs are highly heterogeneous, and our results were based on bulk analysis of CTCs, while previously published data reported a correlation of *AXL* expression with VIM expression in primary tumors.

Tumor progression and therapeutic resistance is not only a matter of cancer cells but also an interplay with the tumor microenvironment (TME) [48]. There is an increased body of evidence that major cellular and non-cellular components of the TME such as regulatory T-cells, dendritic cells (DCs), tumor-associated macrophages (TAMs), natural killer (NK) cells, fibroblasts, and other immune-stromal cells are modified by *AXL* overexpression1 [10,49,50]. A very recent study that is mainly based on animal models has shown that AXL is linked to negative loops of these immune cells and that AXL inhibition could enhance immune responses [51]. Resistance to combined immunotherapy and radiation therapy was related to AXL overexpression in tumors, while sensitivity to immunotherapy was increased through AXL inhibition [52]. In preclinical models, it was found that all tumors expressing *AXL* were also expressing *PD-L1* and thus cooperative targeting boosts antitumor immunity [53]. mRNA profiles from NSCLC datasets revealed correlation also with *CXCR4* and *CXCR6* expressions [54]. High *AXL* expression in the tumor tissue of NSCLC patients revealed predictive significance of survival in patients treated with ICI [55] and after chemotherapy progression [56].

Based on the above evidence that *AXL*-mRNA overexpression plays a key role in the modulation of the immunosuppressive microenvironment, in this study we evaluated for, the first time, *AXL*-mRNA overexpression in enriched CTC fractions of NSCLC patients before and during immunotherapy in NSCLC. According to our findings, *AXL* was overexpressed in enriched CTC fractions in an increased number of NSCLC patients of Group B after three or four cycles of anti-PD-1 therapy compared to baseline; whereas, no *AXL*-mRNA overexpression was detected in enriched CTC fractions at PD analyzed only for 10 patients. However, this is a retrospective analysis and, thus, the small group of NSCLC patients that reached PD may not be representative of the presence of *AXL*-mRNA overexpression at this time point. It is recommended that a larger cohort study could further clarify the implication of *AXL*-mRNA overexpression at disease progression and resistance to immunotherapy.

It is important to highlight that in Group A, *AXL*-mRNA overexpression was detected in 12.8% of patients at baseline and increased to 16.1% after the first cycle, indicating an early adaptive response to osimertinib, while in Group B, *AXL*-mRNA overexpression rose from 6.9% at baseline to 11.3% during immunotherapy. The absence of *AXL*-mRNA overexpression at PD in Group B contrasts sharply with Group A, where 12.5% of PD samples remained *AXL*-positive. This could be due to the very small sample size (*n* = 10) at PD for Group B, or it may indicate a different resistance trajectory under immunotherapy, potentially expected within the broader context of NSCLC heterogeneity [57].

One of the limitations of our study is the significant discrepancy in the size of the two treatment cohorts, (39 patients in the osimertinib group versus 116 patients in the immunotherapy group). This imbalance limits the ability to compare the clinical utility of *AXL* as a biomarker across different treatment modalities. Moreover, the small number of patient samples that we analyzed did not allow us to evaluate the clinical significance based on Kaplan–Meier curves. However, based on our results, on previous evidence and also on the growing interest of the development and efficacy of AXL-inhibitors, we suggest that evaluation of *AXL*-mRNA expression in CTCs is a very promising biomarker that should be further tested in future clinical studies including larger cohorts of patients, since it could serve as a novel therapeutic target during longitudinal monitoring of NSCLC patients for guiding treatment decisions.

Lately, many clinical trials are testing the efficacy of various types of AXL-inhibitors in NSCLC patients as monotherapy or in combination with other agents [8]. The FDA has granted a fast track designation to bemcentinib (BGB324), an oral small molecule that is an inhibitor of AXL kinase, in combination with an anti-PD-L1 agent for patients with STK11 altered advanced or metastatic NSCLC (NCT03184571) [20]. Combinations of bemcentinib with erlotinib (NCT02424617), docetaxel (NCT02922777), and pembrolizumab (NCT03184571) exhibited tolerability in advanced NSCLC. Mecbotamab vedotin (BA3011), a conditionally active biologic anti-AXL antibody drug conjugate (CAB-AXL-ADC) is currently being investigated in combination with PD-1 or PD-L1 inhibitor in NSCLC (phase II, NCT04681131). In the SAFFRON-103 study (NCT03666143), sitravatinib combined with the anti-PD-1 antibody tislelizumab exhibited tolerance and objective responses in patients with advanced NSCLC [58]. Currently, sitravatinib and nivolumab are under evaluation in a phase III trial (NCT04921358) for advanced NSCLC. A novel AXL inhibitor, ONO-7475 combined with osimertinib proved effective to suppress resistance in *EGFR*-mutant NSCLC cells with AXL overexpression [59]. Very recently, the results from phase I/II study of Enapotamab Vedotin, an AXL-specific ADC in solid tumors including NSCLC, showed manageable safety profiles. However, antitumor activity did not show superior clinically meaningful responses as monotherapy compared to other therapies [60].

It is now well established that CTC molecular characterization at the mRNA level has a strong potential not only to provide information on tumor heterogeneity and alternative treatment resistance alterations but also to guide therapeutic interventions in NSCLC [61,62,63]. Based on our findings, we suggest that *AXL*-mRNA overexpression in CTCs should be further evaluated as a potential biomarker for anti-AXL targeted therapies in NSCLC. Considering the recent growing interest in the development and evaluation of new AXL-inhibitors, our observations could support the implementation of larger clinical studies to clarify the role of *AXL*-mRNA overexpression in CTC fractions as a potential novel therapeutic target during longitudinal monitoring of patients with NSCLC.

## 5. Conclusions

Our results indicate that *AXL*-mRNA overexpression in CTC fractions deserves to be further evaluated through larger clinical studies as a potential biomarker for anti-AXL targeted therapies in NSCLC.

## Data Availability

The data presented in this study are available on request from the corresponding authors. The data are not publicly available due to ethical restrictions.

## Abbreviations

The following abbreviations are used in this manuscript:

B2M	Beta-2 microglobulin
CTC	Circulating tumor cells
ctDNA	Circulating tumor DNA
EGFR-TKIs	Epidermal Growth Factor Receptor-Tyrosine Kinase Inhibitors
EMT	Epithelial-to-mesenchymal transition
HD	Healthy donors
ICI	Immune checkpoint inhibitors
NSCLC	Non-small-cell lung cancer
PB	Peripheral blood
PBMC	Peripheral blood mononuclear cells
PD	Progression of disease
TME	Tumor microenvironment
VIM	Vimentin
